# Single application of 4% dimeticone liquid gel versus two applications of 1% permethrin creme rinse for treatment of head louse infestation: a randomised controlled trial

**DOI:** 10.1186/1471-5945-13-5

**Published:** 2013-04-01

**Authors:** Ian F Burgess, Elizabeth R Brunton, Nazma A Burgess

**Affiliations:** 1 Medical Entomology Centre, Insect Research & Development Limited, 6 Quy Court, Colliers Lane, Stow-cum-Quy, Cambridge CB25 9AU, UK

**Keywords:** Dimeticone, Head lice, Insecticides, Pediculosis, Permethrin, Physical mode of action, Treatment regimen

## Abstract

**Background:**

A previous study indicated that a single application of 4% dimeticone liquid gel was effective in treating head louse infestation. This study was designed to confirm this in comparison with two applications of 1% permethrin.

**Methods:**

We have performed a single centre parallel group, randomised, controlled, open label, community based trial, with domiciliary visits, in Cambridgeshire, UK. Treatments were allocated through sealed instructions derived from a computer generated list. We enrolled 90 children and adults with confirmed head louse infestation analysed by intention to treat (80 per-protocol after 4 drop outs and 6 non-compliant). The comparison was between 4% dimeticone liquid gel applied once for 15 minutes and 1% permethrin creme rinse applied for 10 minutes, repeated after 7 days as per manufacturer’s directions. Evaluated by elimination of louse infestation after completion of treatment application regimen.

**Results:**

Intention to treat comparison of a single dimeticone liquid gel treatment with two of permethrin gave success for 30/43 (69.8%) of the dimeticone liquid gel group and 7/47 (14.9%) of the permethrin creme rinse group (OR 13.19, 95% CI 4.69 to 37.07) (p < 0.001). Per protocol results were similar with 27/35 (77.1%) success for dimeticone versus 7/45 (15.6%) for permethrin. Analyses by household gave essentially similar outcomes.

**Conclusions:**

The study showed one 15 minute application of 4% dimeticone liquid gel was superior to two applications of 1% permethrin creme rinse (p < 0.001). The low efficacy of permethrin suggests it should be withdrawn.

**Trial registration:**

Current Controlled Trials ISRCTN88144046.

## Background

In Europe control of head louse infestation is now mainly achieved by use of physically acting preparations [1]. The majority of these are based on the polydimethysiloxane, known as dimeticone. This compound exists in a variety of forms from the volatile hexamethyldisiloxane, with a viscosity of just 0.65 centistokes (cSt) through to high molecular weight gums with a viscosity of several million cSt. The level of polymerisation during manufacture results in a range of materials with such variable physical characteristics that selection of the right molecular weight and viscosity is potentially critical for gaining maximum effectiveness in this application.

The first dimeticone based product was Hedrin 4% lotion, approved as a medicine for sale in the UK in 2005, which used dimeticone of 100K cSt viscosity in a decamethylcyclopentasiloxane (cyclomethicone D5) volatile fluid vehicle. This product is also widely used in Europe, mostly as a class I medical device, and has been shown efficacious in several clinical field studies [2-4]. Since that time variant formulations have been developed in order to improve handling and application of the product and also the cosmetic characteristics.

The original 4% dimeticone lotion is applied for 8 hours or overnight on two occasions a week apart. Subsequent investigations both in the laboratory and in clinical trials have looked at shorter application times for this product and for the variants. One recently described study outcome found that no lice of any development stage were present following the first application of product in a two treatment regimen using a 15 minutes exposure time for a spray gel variant [5]. This report describes the first study investigating the efficacy of a single application regimen for Hedrin 4% dimeticone liquid gel, tested in a comparison trial against two applications of 1% permethrin creme rinse.

## Methods

### Participants

We recruited participants mostly through contact with families who had expressed a wish to be involved in research and advertising on local radio and in parish magazines. An information booklet was sent to each family and an appointment arranged for an investigator to visit. All household members were offered screening for head lice using a standard detection comb (“PDC”, KSL Consulting, Denmark). The intensity of infestation was graded as heavy, medium, or light using criteria employed in previous studies [2-4].

We also used eligibility criteria from previous studies [2-4], excluding those with known sensitivity to treatment components or long terms scalp conditions other than lice, those treated for lice within 2 weeks previously, and who had used hair bleach, dyes, or permanent waves, or had been treated with trimethoprim containing preparations within the last month. Pregnancy, breast feeding, participation in another clinical study within 4 weeks or prior participation in the current study, were also grounds for exclusion. All eligible family members could be enrolled and on agreement were conducted through a standard informed consent and assent procedure. The practical lower age limit was 2 years although approval for the products was as young as 6 months. There was no upper age limit.

Demographic data were collected after consent was taken, including gender, age, hair characteristics, and appointments made for subsequent assessment and treatment visits. No payment was offered for participation. Ineligible household members with lice were offered standard of care treatment using 4% dimeticone liquid gel to minimise reinfestation of study participants.

### Ethics

Ethical approval was granted by Central London Research Ethics Committee 2 (EudraCT 2011-000257-23).

The study was conducted in compliance with Good Clinical Practices, and conformity with the principles of the Declaration of Helsinki and of European Union Directive 2001/20/EC. All participants received study information at least 24 hours before enrolment and all stated that they understood the purpose and requirements of the investigation before giving consent. Parents or guardians gave written consent for children younger than 16 years and children also provided written assent, where able to do so, witnessed by the parent or guardian.

### Treatments

The products used for this randomised controlled trial were different in both appearance and method of application so blinding at the time of treatment was not possible.

4% dimeticone liquid gel (Hedrin Once liquid gel, Thornton & Ross Ltd, Huddersfield, UK) contains 4% high molecular weight dimeticone, 1,6,10-dodecatrien-3-ol,3,7,11-trimethyl, PEG/PPG dimeticone co-polymer, and silica silylate. It was supplied in 150mL polyethylene bottles and applied systematically to dry hair over the whole scalp using the inbuilt dropper cap. In each case the investigators spread the fluid through the hair using their fingers to ensure thorough coverage. The product was applied once for 15 minutes and then washed out using shampoo and water.

1% permethrin creme rinse (Lyclear creme rinse, Omega Pharma UK, London, UK) contains 1% permethrin in a conditioner base with 20% isopropanol. It was supplied in 59mL HDPE bottles with a flip cap. It was applied liberally to shampoo washed and towel dried hair. Investigators ensured that all parts of the hair and scalp were thoroughly coated, which in most cases required more than one bottle. This product was applied for 10 minutes on two occasions 7 days apart followed by rinsing with water only.

In each case parent/guardians were advised of the time to wash the treatments off. Use of nit combs or other pediculicides during the course of the studies was not permitted.

### Outcome measures

The primary outcome measure was elimination of infestation after completion of the treatment regimen. Following treatment on Day 0 we performed follow-ups on Days 1, 6, 9, and 14 by dry detection combing using the “PDC” comb to assess the efficacy of treatment. If any lice were found they were fixed in the case record and the development stage subsequently recorded.

Outcomes of treatment were classified as cure, reinfestation following cure, or treatment failure (with a sub-category for ovicidal failure). Because one of the treatments in this study used one application only we found it necessary to redraft the algorithm used in previous studies for determining outcome criteria.

### Sample size

We anticipated that there was likely to be a disparity in efficacy between dimeticone liquid gel and permethrin creme rinse, based on previous data. Consequently, it was possible that, if different members of a family were randomly allocated different treatments, those receiving the more effective preparation could be reinfested from those receiving the less effective product. Although the protocol allowed for identification of reinfestation at a low level it could not address problems resulting from heavy reinfestation. Therefore a different randomisation model was employed in which all members of a household received the same treatment allocation, with randomisation by family rather than by individual.

An analysis of the variance of the cure rates in relation to household sizes was made using data from previous studies and from this we estimated that the number of participants required using the randomise-by-family approach was higher than the number required using the randomise-by-individual approach (assuming independence and ignoring dropout) by a factor of 1.46.

For a randomise-by-individual approach, assuming a confidence level of 95% and a power of 95%, and conservatively taking the expected cure rates to be 90% for dimeticone liquid gel and 50% for permethrin creme rinse, we estimated the required sample size as 29 participants per treatment. Under the same assumptions, the required number of participants per treatment under the randomise-by-family approach was 42, equivalent to an estimated 22 families per treatment.

### Randomisation and blinding

The randomised treatment allocation sequence was derived using a free to access online computer generated list from http://www.randomization.com (seed 25270, 22^nd^ June 2011). Allocation at the point of delivery was made from instruction sheets enclosed in opaque, sealed, sequentially numbered envelopes distributed to investigators in balanced blocks of eight. Participant families were allocated the next available numbered envelope held by the investigator. Investigators conducting follow up visits were separate from those involved in treatment and thus remained blind of treatment allocation.

### Statistical analysis

We conducted analyses based on both the "intention-to-treat" (ITT) and the "per-protocol" (PP) populations. Differences between groups in baseline characteristics, safety, acceptability, and efficacy were tested using Fisher's exact test for yes/no variables and the Mann–Whitney U test for ranked variables. Where analyses showed important differences in baseline characteristics between the groups, chi-squared and rank tests stratified for these characteristics were conducted and 95% confidence limits presented for the difference between groups in the primary endpoint.

## Results

### Participants

**Figure 1:**
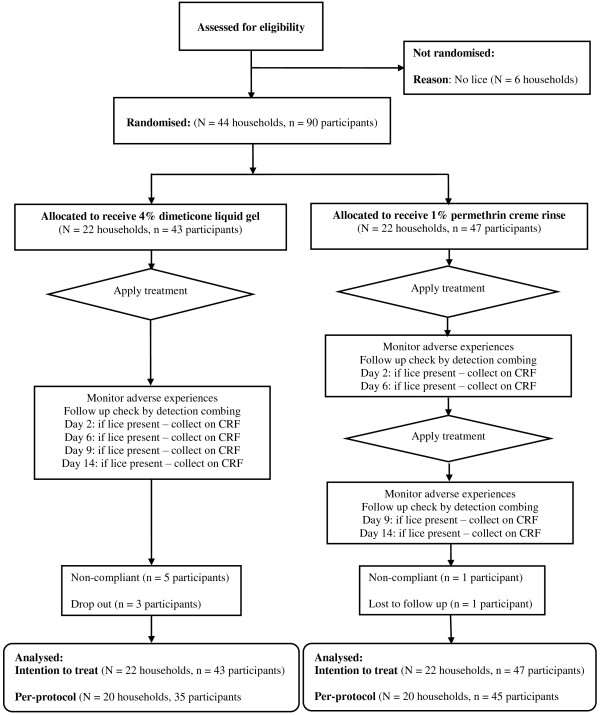
Flowchart of participant progress through the study.

Ninety participants from 44 households were enrolled between 1^st^ July and 3^rd^ November 2011, of whom four participants in two households later dropped out or were lost to follow up (Figure 1). A further six participants completed the study but were non-compliant, either combing out lice (five participants) or out of time on one assessment visit (one participant). Therefore the ITT population analysed was 90, but as a result of the protocol violations the per-protocol population was 80 across 40 households broken down as follows: dimeticone liquid gel = 35 participants from 20 households; permethrin creme rinse = 45 from 20 households.

Baseline characteristics were recorded for all participants at Day 0 of whom 73 (81.1%) were female and ranged from 2 to 45 years in age, mean age 11.7 years. The 44 households, varied in size from 2 to 9 occupants (mean 4.39). Numbers of participants per household were: 1 (17 households), 2 (16 households), 3 (6 households), 4 (3 households), 5 or 6 participants (1 household each). Of the 90 participants, the initial infestation was classified as “light” in 52, “moderate” in 17 and “heavy” in 21. There was no difference between treatments in this characteristic.

Consistent with the large proportion of females in the study, the proportion with hair “ears to shoulders” or “below shoulders” was very high (89.3% in the dimeticone group and 84.9% in the permethrin group). There were similarly high numbers of participants with “thick” hair (61.7% and 53.8% respectively). Both groups had approximately 37% of participants with “wavy” or “slightly curly” hair, which is a recently observed increased trend toward “wavy” hair compared with older studies. Only a small proportion of participants had hair that was dry (7.4%) or oily (8.1%).

### Outcomes

The Day 1 analyses included all 90 participants; Day 6 analyses related to 89 participants following a drop out, with a subsequent drop out of 2 other participants from the same household so that 87 participants remained on Day 9. The Day 14 analyses included 86 participants due to one lost to follow up, with exclusion of a further 6 participants from the PP analyses for protocol violations.

For the intention to treat outcomes by individual, we conducted an endpoint analysis of rate of cure (or exceptionally cure followed by reinfestation as defined by algorithm) in the 90 participants in the ITT population. According to these criteria, success was achieved overall by 30/43 (69.8%) for dimeticone liquid gel group (26 cure, 4 reinfestation) and by 7/47 (14.9%) for the permethrin creme rinse group (6 cure, 1 reinfestation). The difference in rate of success between the dimeticone and permethrin groups was estimated as 54.9% (95% CI of 35% to 75%) (OR 13.19, 95% CI 4.69 to 37.07) which meant that there was a highly significant (p < 0.001) superiority of dimeticone liquid gel compared with permethrin creme rinse in the population tested. Dimeticone liquid gel not only showed a high ovicidal effect, as judged by not finding young nymphs following the single application of the product (32 participants), but also a high proportion were entirely louse free throughout (the “cure” group of 26 participants) (Table 1). In contrast, nearly all participants in the permethrin group were found to have newly hatched nymphs at some point in the study, showing the product has a low capacity to inhibit eggs from hatching in addition to any impact on efficacy resulting from insecticide resistance (Table 2).

**Table 1:** Comparison of outcomes by participant – 4% dimeticone versus 1% permethrin

**Outcome measurement**	**4% Dimeticone**		**1% Permethrin**		**P value**
**ITT analysis**					
Number of households	22		22		
Number of participants	43		47		
Cure or cure followed by reinfestation	30	69.8%	7	14.9%	< 0.001
Relative success rate (95% CI)			4.68 (2.30 – 9.35)		
Cure	26	60.5%	6	12.8%	< 0.001
Inhibition of egg hatching	32	74.4%	6	12.8%	< 0.001
**PP analysis**					
Number of households	20		20		
Number of participants	35		45		
Cure or cure followed by reinfestation	27	77.1%	7	15.6%	< 0.001
Relative success rate (95% CI)			4.96 (2.45 – 10.03)		
Cure	26	71.4%	6	13.3%	< 0.001
Inhibition of egg hatching	30	85.7%	6	10.6%	< 0.001

**Table 2:** Numbers of lice recovered – 4% dimeticone versus 1% permethrin

**Endpoint**	**Lice collected**			
	**Day 1**	**Day 6**	**Day 9**	**Day 14**
**4% Dimeticone liquid gel**				
Number of participants combed	43	42	40	40
Number of participants with lice	3	4	8	12
Total lice removed	3	27	24	45
Stage 1 nymphs removed	1	5	5	2
Stage 2 nymphs removed	2	18	8	10
Stage 3 nymphs removed	0	2	7	7
Adult males removed	0	0	2	9
Adult females removed	0	2	2	17
**Participants louse free (%) ITT**	**93.0%**	**90.5%**	**80.0%**	**70.0%**
**Participants louse free (%) PP**	**97.1%**	**94.3%**	**82.9%**	**77.1%**
**1% Permethrin creme rinse**				
Number of participants combed	47	47	47	46
Number of participants with lice	29	37	30	38
Total lice removed	359	741	259	375
Stage 1 nymphs removed	222	405	63	40
Stage 2 nymphs removed	18	204	46	51
Stage 3 nymphs removed	24	67	81	54
Adult males removed	23	17	27	91
Adult females removed	72	48	42	139
**Participants louse free (%) ITT**	**38.3%**	**21.3%**	**36.2%**	**17.4%**
**Participants louse free (%) PP**	**37.8%**	**20.0%**	**37.8%**	**17.7%**

We obtained per-protocol outcomes by individual by elimination of protocol violators from the analysis to give PP success rates of 27/35 (77.1%) for the dimeticone liquid gel group (25 cure, 2 reinfestation) and 7/45 (15.6%) for the permethrin creme rinse group (6 cure, 1 reinfestation), giving an advantage of 61.6% (95% CI, 40% to 80%) (OR 18.32, 95% CI 5.93 to 56.6) for the dimeticone treatment over permethrin, which was also highly significant (p < 0.001).

Using the same approach in the analysis of outcome by household, the advantage to dimeticone liquid gel was similar (p < 0.01 or p < 0.001) for all the outcomes, equivalent to a relative total success rate of 6.50 (95% confidence interval (CI) 1.66 to 25.5), or an odds ratio (OR) of 14.4 (95% CI 2.68 to 77.8), reflecting the difference of risk for a whole household compared with that for an individual.

All participants received the required number of treatments. Dimeticone liquid gel was given once, and the mean total product used was 63.91g (equivalent approximate cost = €6.20-€7.40). Permethrin creme rinse was given twice, and each treatment often involved two bottles and occasionally three. Over the two treatments combined, the mean number of bottles of creme rinse used per family member was 3.31, and the mean total product used, 141.58g (equivalent approximate cost = €8.30-€16.60), which was significantly (p < 0.001) more than for dimeticone.

### Adverse events

Members of seven families experienced one or more adverse events, nine people treated with permethrin and eight with dimeticone. Only two people had an adverse event that was considered possibly related to treatment. One was a rash on the back of the neck following each treatment with permethrin creme rinse and the other, dry skin, following dimeticone liquid gel. There were no serious adverse events.

## Discussion

It has long been considered that longer application times when using head louse treatment lotions are more effective than shorter applications. This was demonstrated *in vitro* using insecticide based products in which the formulation vehicle evaporates and concentrates the active material on the louse surface. The same approach was initially taken with physically acting preparations such as 4% dimeticone lotion and preliminary results from a first small study did indicate that an 8 hour or overnight treatment was more effective than one of only 20 minutes [2]. However, in a later study we found that, contrary to expectation, reducing the application time for 4% dimeticone spray gel to 15 minutes not only increased the effectiveness but appeared to be wholly effective with a single application [5].

Although the majority action of 4% dimeticone lotion is derived from the dimeticone being deposited in the insect spiracular and tracheal systems, resulting in blocking of water excretion [6], it is necessary for it to be carried there by the solvent component of the product. If the solvent does not deliver sufficient dimeticone to the target site the activity may be reduced.

The perceived advantage of the 4% dimeticone gel over the original 4% lotion is that the gel seems to adhere to the lice more effectively and, in the spray form, is easier to direct and control ensuring all lice are killed and those eggs close to hatching are inhibited from doing so, as was demonstrated in our earlier study in which the product gave 100% efficacy after the first application in that two application trial [5]. In this study, most lice found by investigators during follow up assessments could be attributed to eggs that hatched after treatment, presumably because they had been missed as a result of inadequate spreading of the liquid gel using the dropper bottle. Some of the children had also been observed to present with hair dampened by sweat at the time of the treatment application, which may have acted as a barrier to the silicones forming a complete coating on some louse eggs, with the possible result that silicone was not able to reach the vulnerable surfaces of the eggs due to the presence of a water film. The four infestations in the ITT group without nymphs were all attributable to reinfestation from contacts either within the family or the local community.

This randomised controlled trial has clearly demonstrated a difference in overall activity between a physically acting preparation using a single application and two applications of an insecticide based product affected by insecticide resistance. Hitherto, the majority of claims that different products containing silicones, and other physically acting preparations, are effective with a single application have not been based on published clinical data but have been extrapolations either from *in vitro* data or else obtained in studies where the products were applied twice. This study has not only confirmed efficacy for a single application of Hedrin 4% dimeticone liquid gel, and demonstrated a difference in overall activity between a physically acting preparation and an insecticide based product affected by insecticide resistance, but has also demonstrated that extrapolations about treatment outcomes may be misleading, in the absence of clinical confirmation.

This trial has also provided the first clear evidence that louse eggs do not all hatch within 7 days (Table 2). We and many other investigators in the past had assumed this hatching period primarily because previously we had no other experimental evidence upon which to base a conclusion. We found from our earlier double application study [5] that this does not necessarily constitute a significant problem for treatment because any lice hatching from eggs after a first treatment appear to be killed by a second treatment at 7 days. A treatment given at that time delivers more of the active material to any unhatched eggs, which further inhibits their chances of hatching. However, this information that eggs can take longer than one week to hatch emphasises the need for vigilance in confirming whether a treatment has been effective, by detection combing after completion of the treatment regimen, and also confirms the need for adequate application technique [7]. So, just because more than 90% of people appear louse free 2 days after a second treatment it does not mean that further checks are not necessary as some viable eggs may remain.

Although numerous lice were eliminated using the 1% permethrin creme rinse at each of the treatment applications, more than 60% of participants received no respite from infestation and, of those who appeared louse free one or two days after treatment, fewer than half were cured of their infestation, even though we used more than twice the supposedly adequate application dose on each participant. Why then did we choose 1% permethrin as the comparator product rather than some alternative preparation? The reason 1% permethrin was used in this study was to provide data in comparison with a known medicinal product for those territories where such data are required by decision makers before issuing approvals. On this basis the number of possible comparators is extremely limited because most products have a limited geographic distribution. The only other insecticide active used widely is 0.5% malathion but this was not selected because the range of formulations used in different territories are not comparable even within themselves (shampoos, alcohol based lotions with or without terpenoids added, and water based emulsions). Additionally, the one product still listed in the UK was not available at the time the study was conducted.

The outcome using permethrin mirrors closely the findings of a previous investigation comparing treatments using this product with and without combing [8], and also reflects the outcome found in other studies. Given the number of investigations in which permethrin and other insecticide products have delivered an unacceptably low efficacy [3,8-12], a review of the licensing of these preparations appears long overdue. It is understandable that sponsors may wish to use insecticide based products as comparators in clinical studies because they have a long history of use and are familiar to the decision makers within the various health services where the profile of new products needs to be raised. However, we have found that ethics review boards are increasingly reluctant to authorise their use in clinical investigations primarily because their efficacy is not much greater than using a placebo. Therefore, as long as these products retain a marketing authorisation, and the validity of this is not reviewed by the various competent authorities, consumers and prescribers will continue to use ineffective products. Consequently, now is probably the time for such a review to take place and for insecticide products to be removed from the market simply because so many treatments fail.

## Conclusions

This study has shown that a single application of 4% dimeticone liquid gel is effective to eliminate head louse infestation and that the higher viscosity of this product allows this to be achieved using a treatment time of 15 minutes. However, as with all treatments, it is possible to miss some louse eggs during treatment, requiring post treatment vigilance for emerging nymphs more than 7 days after treatment.

## Competing interests

IFB has been a consultant to Thornton & Ross Ltd, the sponsor of this study and manuscript, and to various other makers of pharmaceutical products, medical devices, and combs for treating infestations of head lice and their eggs. ERB and NAB declare no competing interests in this work.

## Authors’ contributions

IFB, ERB, NAB collectively conceived, designed, and carried out the investigation. IFB performed some analyses of the data and wrote the draft manuscript. ERB managed the documentation of the study. NAB collected data. All authors read and approved the final manuscript.

## Pre-publication history

The pre-publication history for this paper can be accessed here:

http://www.biomedcentral.com/1471-5945/13/5/prepub
